# Supplementary material for: Citrullinated ENO1 Vaccine Enhances PD-1 Blockade in Mice Implanted with Murine Triple-Negative Breast Cancer Cells
Source: Vaccines (Basel). 2025 Jun 11;13(6):629. doi: 10.3390/vaccines13060629 (PMC12197626; doi:10.3390/vaccines13060629)

CPC scientific

Certificate of Analysis

**Product Name:**	Peptide 1
**Product #:**	951203 **Lot #:** CV-10-00443
**Sequence:**	Arg-Ala-Ala-Val-Pro-Ser-Gly-Ala-Ser-Thr-Gly-Ile-Tyr-Glu-Ala-Leu-Glu-Leu-Arg-Asp-Asn-Asp-Lys-Thr-Arg
**Molecular Weight:**	2691.0
**Mass Spectral Analysis:**	Electrospray "Exhibits correct MW" (see attached MS spectrogram)
**HPLC Analysis:**	Peptide purity: 99.3% (see attached RP-HPLC chromatogram)
**Solubility:**	1 mg/ml in water
**Appearance:**	White lyophilized powder
**Counter Ion:**	Trifluoroacetate

Cert. of Analysis Remarks:

Remarks: **Not for human use, research purpose only.**

Quality Assurance By: I.y. Wu **Date: 06/01/23**
Quality Control Department

CPC Scientific Inc.
160 E. Tasman Dr., Suite 200 San Jose, CA 95134 USA
Tel: 1-408-734-3800 or 1-877-272-7241 (Toll-free)
Fax: 1-408-734-3810 or 1-877-272-7244 (Toll-free)
E-mail: sales@cpcscientific.com

Injection Data File Directory /2022/2021062802/Results/221102/001.rslt

Sample Description Sample ID:951203 Lot#:CV-10-00443
Mobie Phase:A:0.1%TFA in H2O
B:0.09%TFA in (80%ACN+20%H20)
Flow:1.0ml/min 25.0%-45.0% B buffer in 10min
Column:Phenomenex-C18(2) luna 3um 100A 4.6*100mm A2118#

Injection Acquired Date	11/2/2022	Sample Vial Number	P1-A4
Injection Acquired By	sherry song	Injection Volume	12.000
Instrument Name	2021062802	Injection Volume Unit	µL

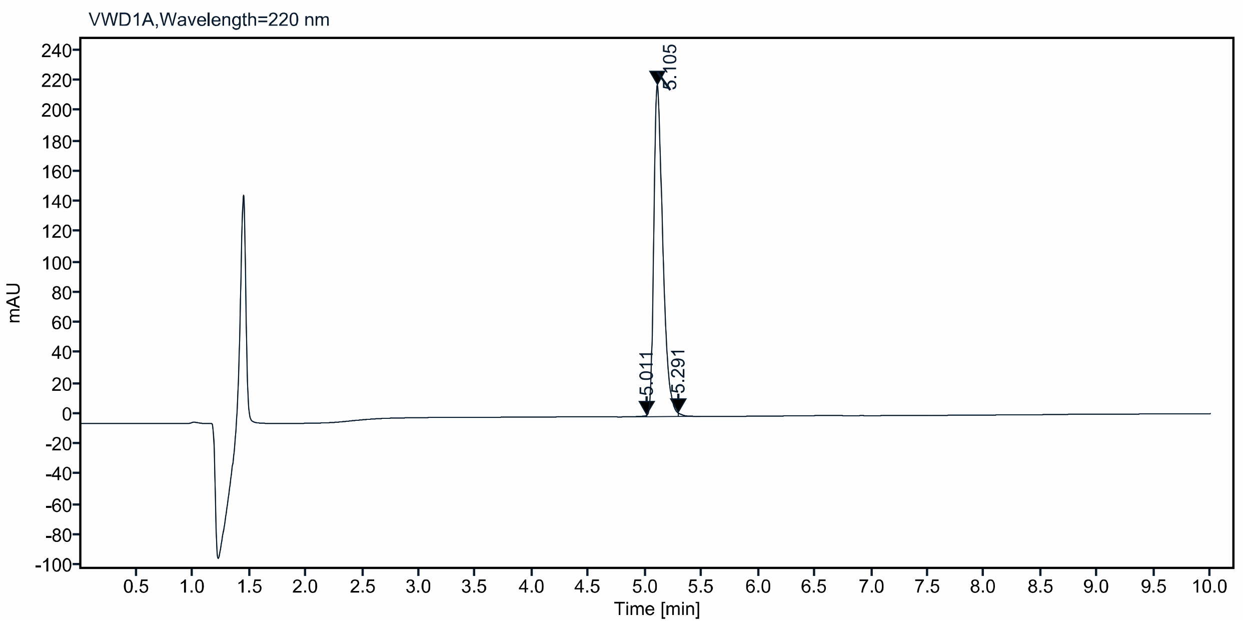

Signal: VWD1A,Wavelength=220 nm

RT [min]	Type	Width [min]	Height	Area	Area%
5.011	BV	0.213	0.927	2.293	0.186
5.105	VF	0.280	218.501	1223.227	99.326
5.291	VV	0.195	2.370	6.010	0.488

951203_221102153143
Type: Unknown ID: 1 Row: 1
Sample Name:
Sample Name: 951203
Lot#: CV-10-00443
Sample Style: Final
Operator: ZWY
Path: E:/newdata/202211
Instrument Method: E:\method\MS-290-160C.meth

951203_221102153143 #6-7 RT: 0.13-0.15 AV: 2 NL: 2.64E6
T: + c ESI Full ms [290.00-2000.00]

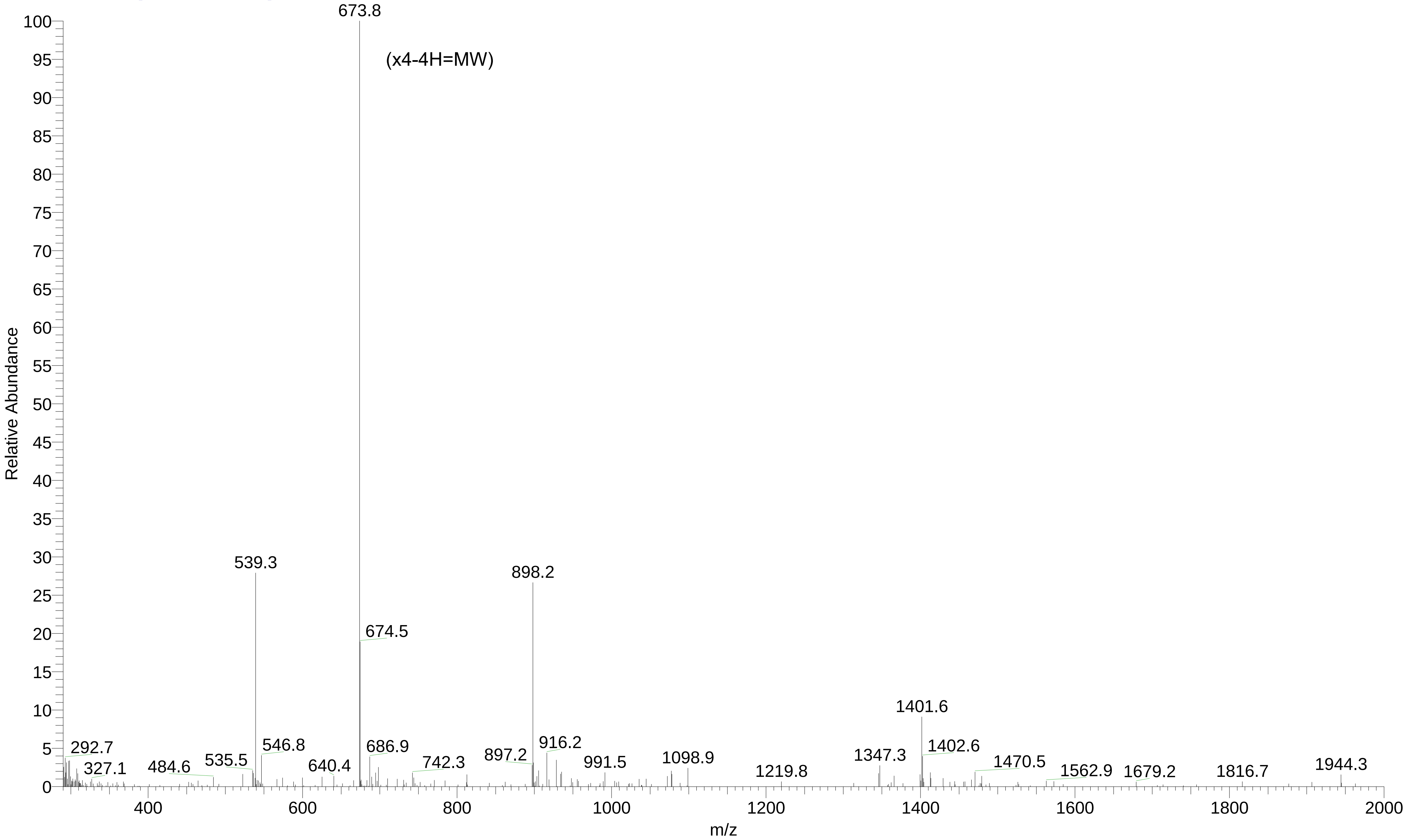

Certificate of Analysis

**Product Name:**	Peptide 2		
**Product #:**	951204	**Lot #:**	CW-05-01542
**Sequence:**	Cit-Ala-Ala-Val-Pro-Ser-Gly-Ala-Ser-Thr-Gly-Ile-Tyr-Glu-Ala-Leu-Glu-Leu-Cit-Asp-Asn-Asp-Lys-Thr-Cit		
**Molecular Weight:**	2693.9		
**Mass Spectral Analysis:**	Electrospray "Exhibits correct MW" (see attached MS spectrogram)		
**HPLC Analysis:**	Peptide purity: 90.7% (see attached RP-HPLC chromatogram)		
**Solubility:**	1 mg/ml in water		
**Appearance:**	White lyophilized powder		
**Counter Ion:**	Trifluoroacetate		

Cert. of Analysis Remarks:

Remarks: **Not for human use, research purpose only.**

Iy. Wu

Quality Assurance By: ______________________________ **Date: 06/14/23**

Quality Control Department

CPC Scientific Inc.
160 E. Tasman Dr., Suite 200 San Jose, CA 95134 USA
Tel: 1-408-734-3800 or 1-877-272-7241 (Toll-free)
Fax: 1-408-734-3810 or 1-877-272-7244 (Toll-free)
E-mail: sales@cpcscientific.com

Injection Data File Directory	/2023/2021062802/Results/230614/001.rslt
Sample Description	Sample ID:951204 Lot#:CW-05-01542<br>Mobie Phase:A:0.1%TFA in H2O<br>B:0.09%TFA in (80%ACN+20%H20)<br>Flow:1.0ml/min 26.0%-46.0% B buffer in 10min<br>Column:Phenomenex-C18(2) luna 3um 100A 4.6*100mm A2016# 50C

Injection Acquired Date	6/14/2023	Sample Vial Number	P1-A6
Injection Acquired By	sherry.song	Injection Volume	10.000
Instrument Name	2021062802	Injection Volume Unit	µL

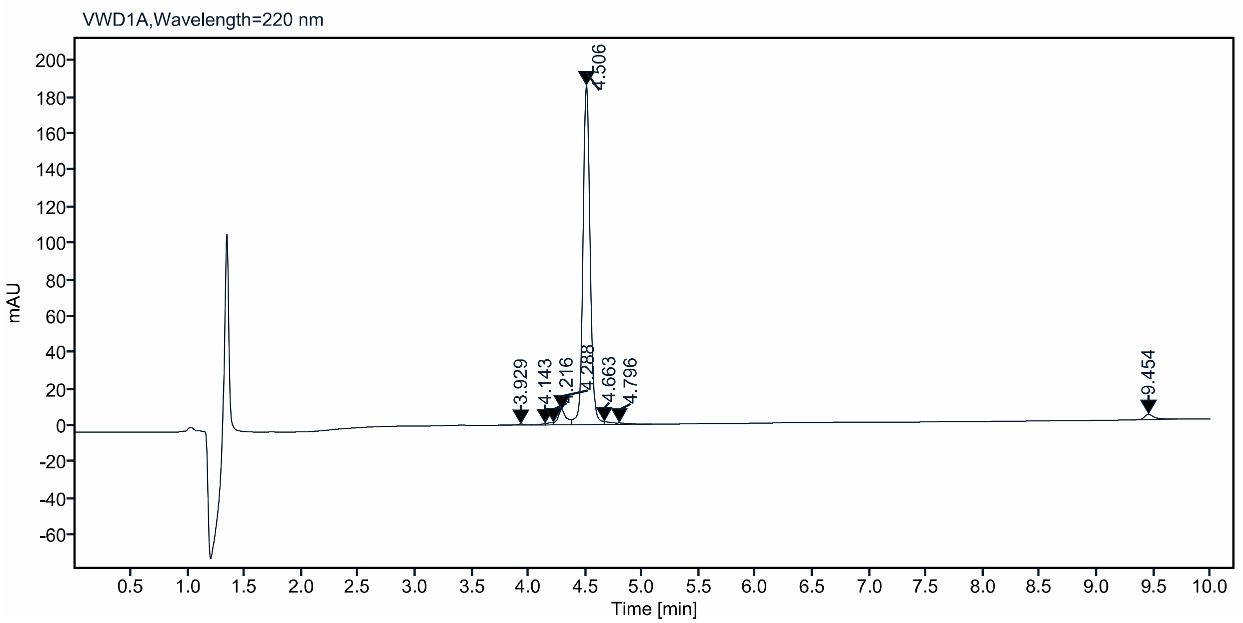

Signal: VWD1A,Wavelength=220 nm

RT [min]	Type	Width [min]	Height	Area	Area%
3.929	BV	0.278	0.554	3.053	0.342
4.143	VV	0.122	0.679	1.967	0.220
4.216	VV	0.073	1.256	4.500	0.504
4.288	VV	0.159	8.314	42.308	4.738
4.506	VF	0.288	185.639	810.049	90.712
4.663	VV	0.107	1.624	7.245	0.811
4.796	VV	0.310	0.798	6.573	0.736
9.454	BV	0.402	2.883	17.297	1.937

Data file: D:\DATE\230614\230614-1 2023-06-14 09-04-10\951204 F.D 6/14/2023 2:07:25 PM

Sample name: 951204

Description: Lot#:CW-05-01542
Sample Type:Final

Acq. method: 290- 2000.M

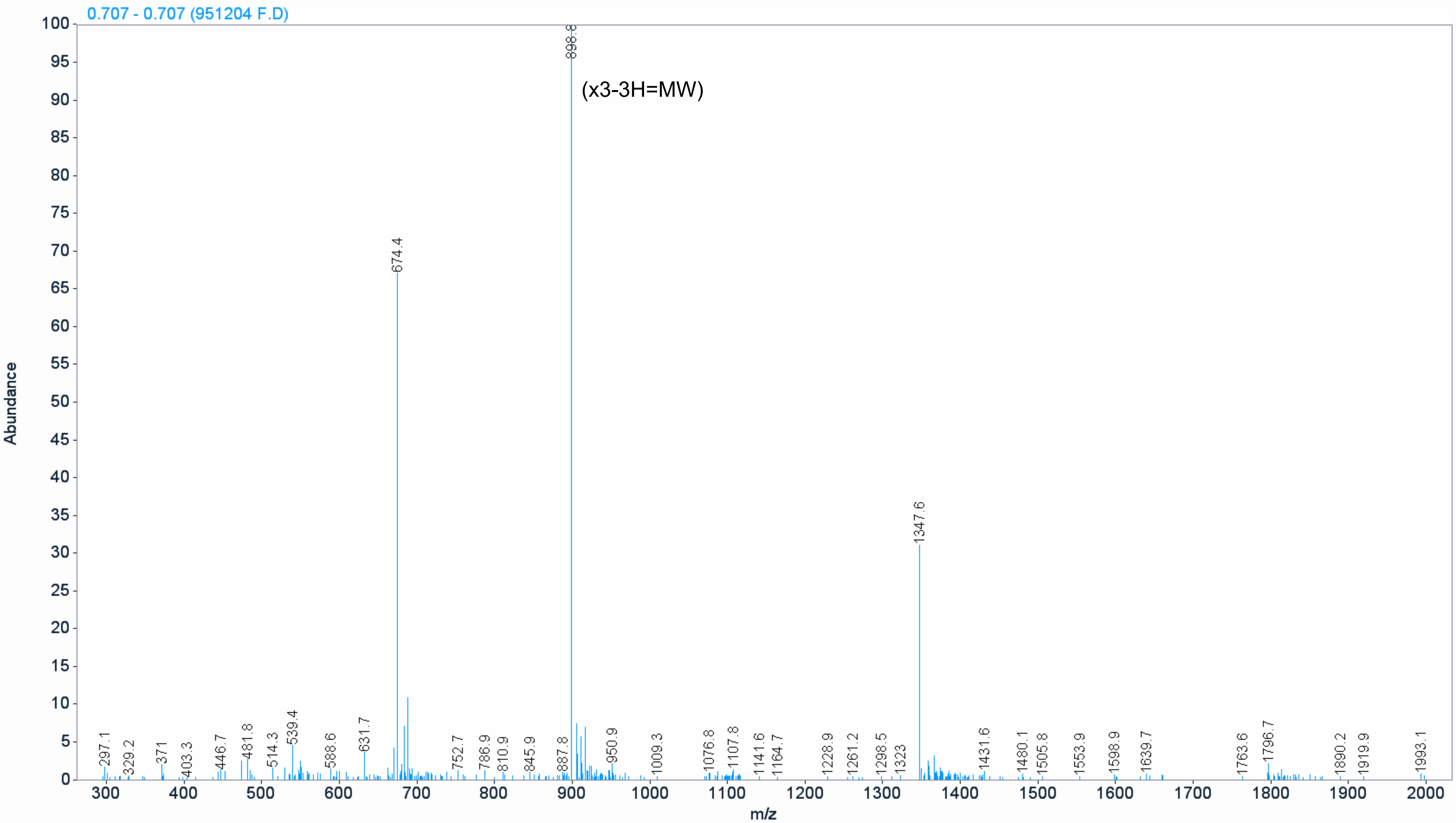

Certificate of Analysis

**Product Name:**	Peptide 3		
**Product #:**	951205	**Lot #:**	CV-10-00444
**Sequence:**	Ala-Met-Gln-Glu-Phe-Met-Ile-Leu-Pro-Val-Gly-Ala-Ser-Ser-Phe-Arg-Glu-Ala-Met-Arg-Ile-Gly-Ala		
**Molecular Weight:**	2513.0		
**Mass Spectral Analysis:**	Electrospray "Exhibits correct MW" (see attached MS spectrogram)		
**HPLC Analysis:**	Peptide purity: 93.6% (see attached RP-HPLC chromatogram)		
**Solubility:**	1 mg/ml in DMSO		
**Appearance:**	White lyophilized powder		
**Counter Ion:**	Trifluoroacetate		

Cert. of Analysis Remarks:

Remarks: **Not for human use, research purpose only.**

Quality Assurance By: I.Y. Wu **Date: 06/01/23**

Quality Control Department

CPC Scientific Inc.
160 E. Tasman Dr., Suite 200 San Jose, CA 95134 USA
Tel: 1-408-734-3800 or 1-877-272-7241 (Toll-free)
Fax: 1-408-734-3810 or 1-877-272-7244 (Toll-free)
E-mail: sales@cpcscientific.com

Injection Data File Directory	/2022/2021062802/Results/221104/002.rslt
Sample Description	Sample ID:951205 Lot#:CV-10-00444<br>Mobie Phase:A:0.1%TFA in H2O<br>B:0.09%TFA in (80%ACN+20%H20)<br>Flow:1.0ml/min 38.0%-58.0% B buffer in 10min<br>Column:Phenomenex-C18(2) luna 3um 100A 4.6*100mm A2118# 50C

Injection Acquired Date	11/4/2022	Sample Vial Number	P1-A1
Injection Acquired By	sherry song	Injection Volume	12.000
Instrument Name	2021062802	Injection Volume Unit	µL

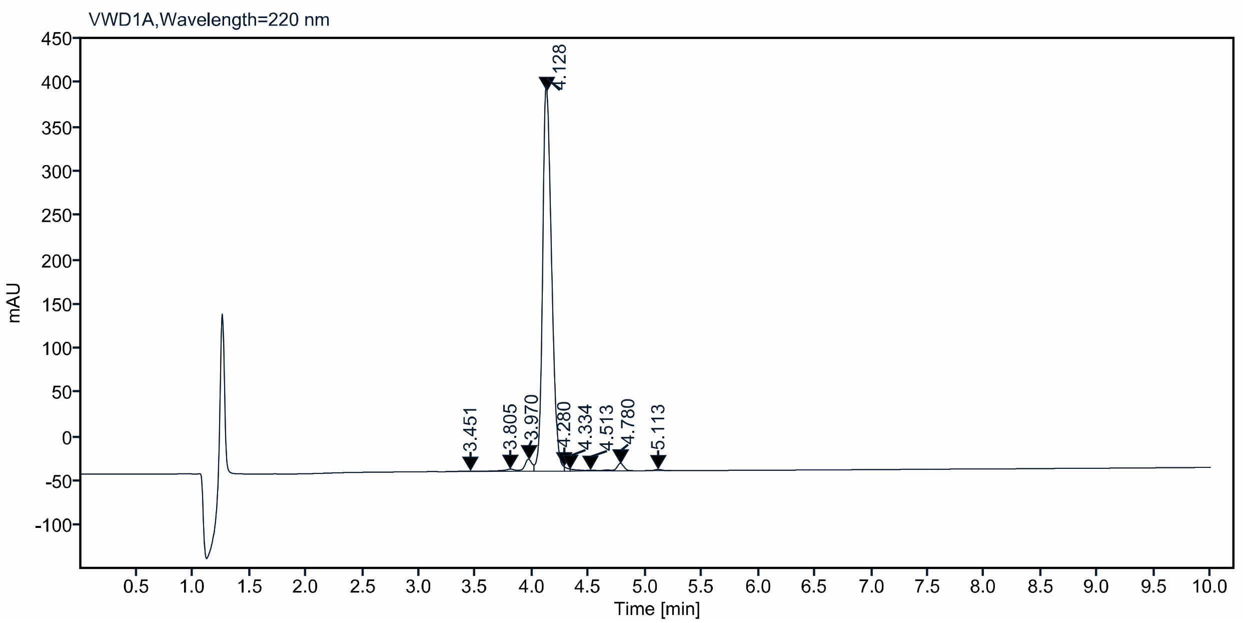

Signal: VWD1A,Wavelength=220 nm

RT [min]	Type	Width [min]	Height	Area	Area%
3.451	BV	0.291	0.587	6.259	0.244
3.805	VV	0.349	2.525	22.909	0.893
3.970	VV	0.133	13.188	59.477	2.317
4.128	VF	0.267	429.151	2404.099	93.660
4.280	VF	0.053	5.140	10.645	0.415
4.334	VF	0.133	2.160	10.130	0.395
4.513	VV	0.122	1.032	5.822	0.227
4.780	VBA	0.355	7.965	41.253	1.607
5.113	BBA	0.227	1.352	6.251	0.244

951205

Type: Unknown ID: 1 Row: 1

Sample Name:
Sample Name: 951205
Lot#: CV-10-00444
Sample Style: Final
Operator: ZWY
Path: E:\newdata\202211
Instrument Method: E:\method\MS-290.meth

951205 #6-7 RT: 0.12-0.14 AV: 2 NL: 1.00E8
T: + c ESI Full ms [290.00-2000.00]

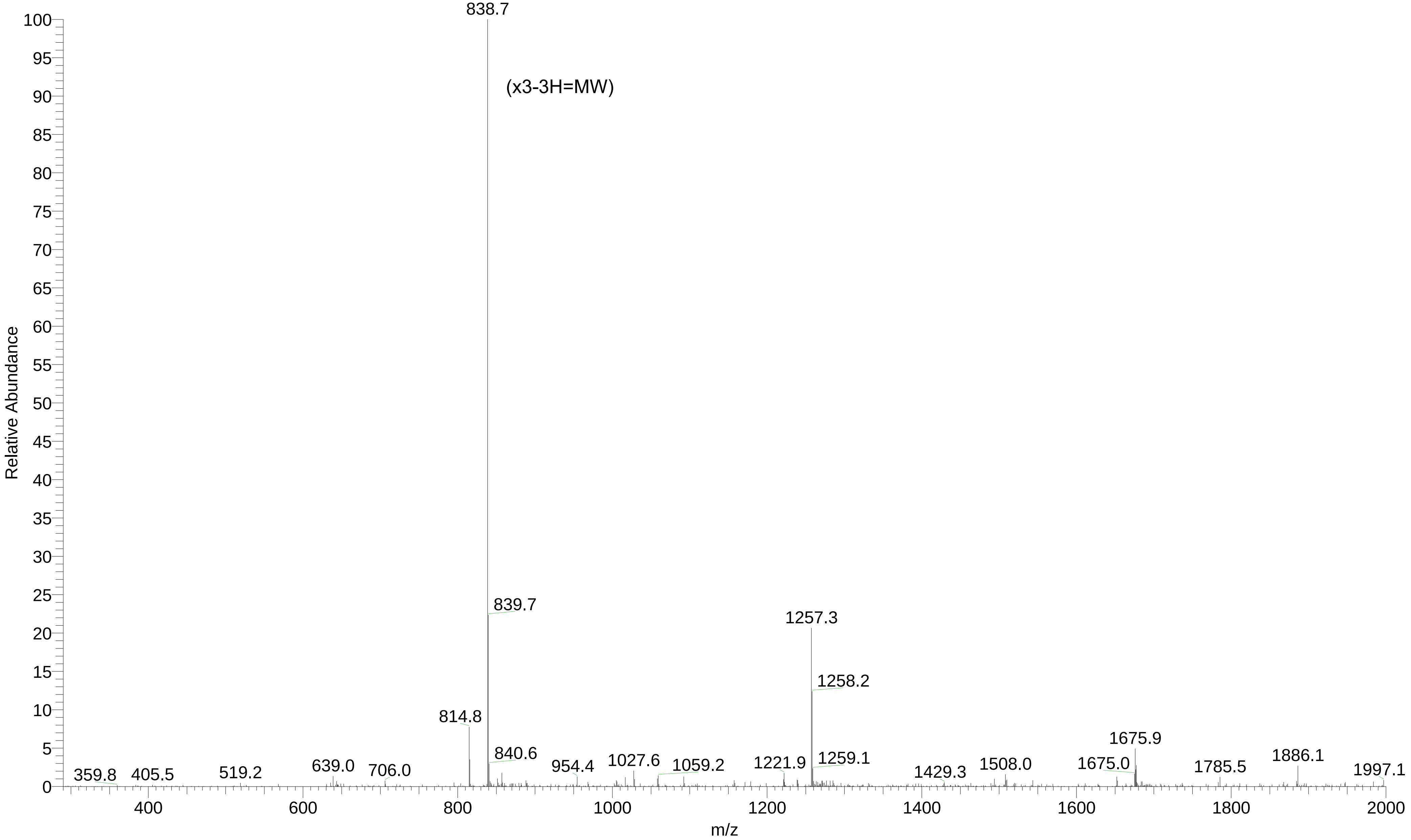

Certificate of Analysis

**Product Name:**	Peptide 4
**Product #:**	951206 **Lot #:** CW-05-01544
**Sequence:**	Ala-Met-Gln-Glu-Phe-Met-Ile-Leu-Pro-Val-Gly-Ala-Ser-Ser-Phe-Cit-Glu-Ala-Met-Cit-Ile-Gly-Ala
**Molecular Weight:**	2515.0
**Mass Spectral Analysis:**	Electrospray “Exhibits correct MW” (see attached MS spectrogram)
**HPLC Analysis:**	Peptide purity: 90.1% (see attached RP-HPLC chromatogram)
**Solubility:**	1 mg/ml in Formic acid
**Appearance:**	White lyophilized powder
**Counter Ion:**	Trifluoroacetate

Cert. of Analysis Remarks:

Remarks: **Not for human use, research purpose only.**

Quality Assurance By: Iay. Wu **Date: 06/07/23**

Quality Control Department

CPC Scientific Inc.
160 E. Tasman Dr., Suite 200 San Jose, CA 95134 USA
Tel: 1-408-734-3800 or 1-877-272-7241 (Toll-free)
Fax: 1-408-734-3810 or 1-877-272-7244 (Toll-free)
E-mail: sales@cpcscientific.com

Injection Data File Directory	/2023/2021062802/Results/230607/001.rslt
Sample Description	Sample ID:951206 Lot#:CW-05-01544<br>Mobie Phase:A:0.1%TFA in H2O<br>B:0.09%TFA in (80%ACN+20%H20)<br>Flow:1.0ml/min 39.0%-59.0% B buffer in 10min<br>Column:Phenomenex-C18(2) luna 3um 100A 4.6*100mm A2016#

Injection Acquired Date	6/7/2023	Sample Vial Number	P1-A1
Injection Acquired By	sherry.song	Injection Volume	10.000
Instrument Name	2021062802	Injection Volume Unit	µL

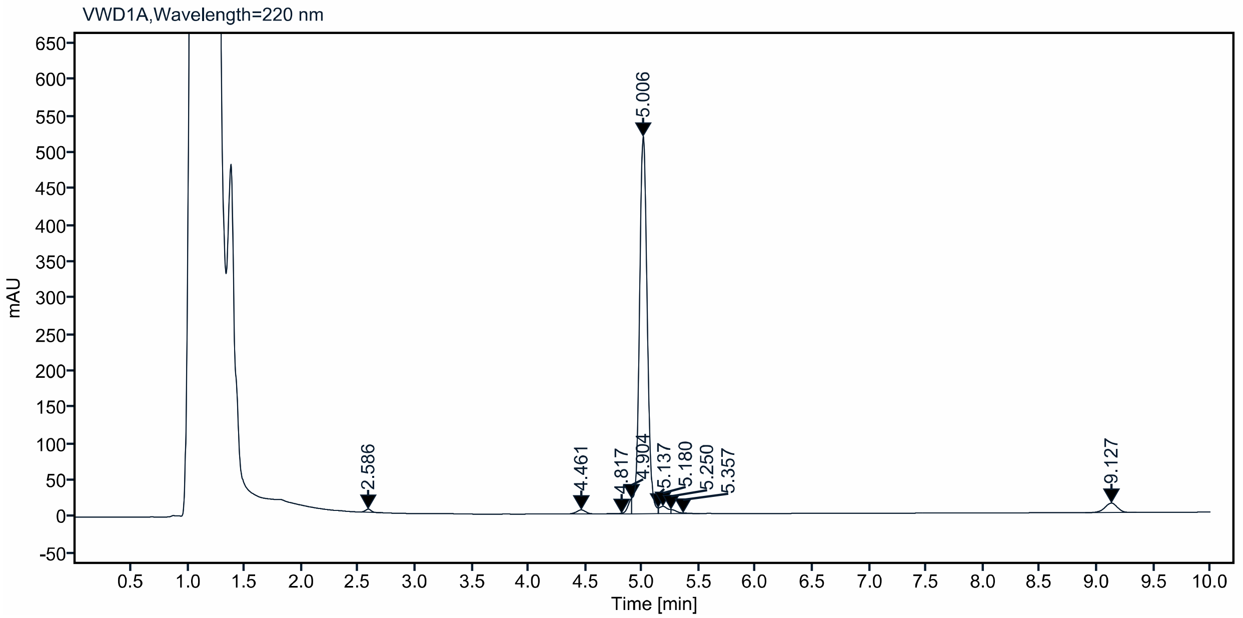

Signal: VWD1A,Wavelength=220 nm

RT [min]	Type	Width [min]	Height	Area	Area%
2.586	BBA	0.178	4.302	15.118	0.552
4.461	BBA	0.273	5.593	30.840	1.126
4.817	BV	0.193	0.802	3.320	0.121
4.904	VV	0.087	21.001	44.744	1.634
5.006	VF	0.233	517.400	2467.219	90.111
5.137	VV	0.002	7.861	1.158	0.042
5.180	VF	0.111	10.114	54.019	1.973
5.250	VF	0.107	5.623	19.769	0.722
5.357	VV	0.143	0.899	3.021	0.110
9.127	BBA	0.448	12.884	98.784	3.608

Data file: D:\DATE\230607\230607-1 2023-06-07 09-34-01\951206F.D 6/7/2023 11:04:02 AM
Sample name: 951206
Description: Lot#:CW-05-01544
Sample Type:Final
Acq. method: 290- 2000.M

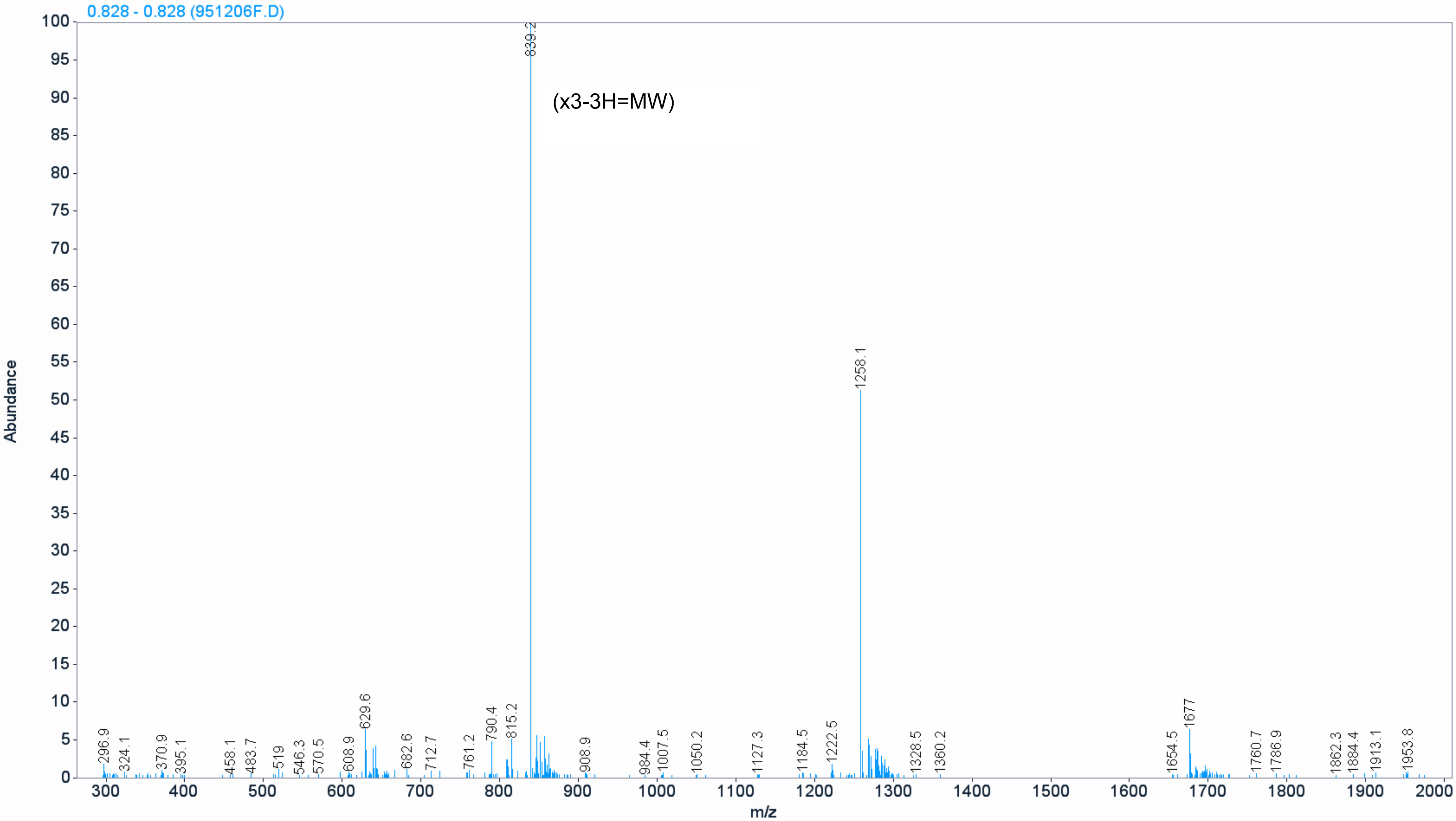

Certificate of Analysis

**Product Name:**	Peptide 5		
**Product #:**	951207	**Lot #:**	CV-11-00091
**Sequence:**	Lys-Ser-Pro-Asp-Asp-Pro-Ser-Arg-Tyr-Ile-Thr-Pro-Asp-Gln-Leu-Ala-Asp-Leu-Tyr-Lys-Ser-Phe-Val		
**Molecular Weight:**	2655.9		
**Mass Spectral Analysis:**	Electrospray "Exhibits correct MW"<br>(see attached MS spectrogram)		
**HPLC Analysis:**	Peptide purity: 93.6%<br>(see attached RP-HPLC chromatogram)		
**Solubility:**	1 mg/ml in water		
**Appearance:**	White lyophilized powder		
**Counter Ion:**	Trifluoroacetate		

Cert. of Analysis Remarks:

Remarks: **Not for human use, research purpose only.**

Quality Assurance By: ______________________ **Date: 06/01/23**

Quality Control Department

CPC Scientific Inc.
160 E. Tasman Dr., Suite 200 San Jose, CA 95134 USA
Tel: 1-408-734-3800 or 1-877-272-7241 (Toll-free)
Fax: 1-408-734-3810 or 1-877-272-7244 (Toll-free)
E-mail: sales@cpcscientific.com

Injection Data File Directory	/2022/2021062802/Results/221111/004.rslt
Sample Description	Sample ID:951207 Lot#:CV-11-00091<br>Mobie Phase:A:0.1%TFA in H2O<br>B:0.09%TFA in (80%ACN+20%H20)<br>Flow:1.0ml/min 35.0%-55.0% B buffer in 10min<br>Column:Phenomenex-C18(2) luna 3um 100A 4.6*100mm A2118# 50C

Injection Acquired Date	11/11/2022	Sample Vial Number	P1-A7
Injection Acquired By	sherry song	Injection Volume	12.000
Instrument Name	2021062802	Injection Volume Unit	µL

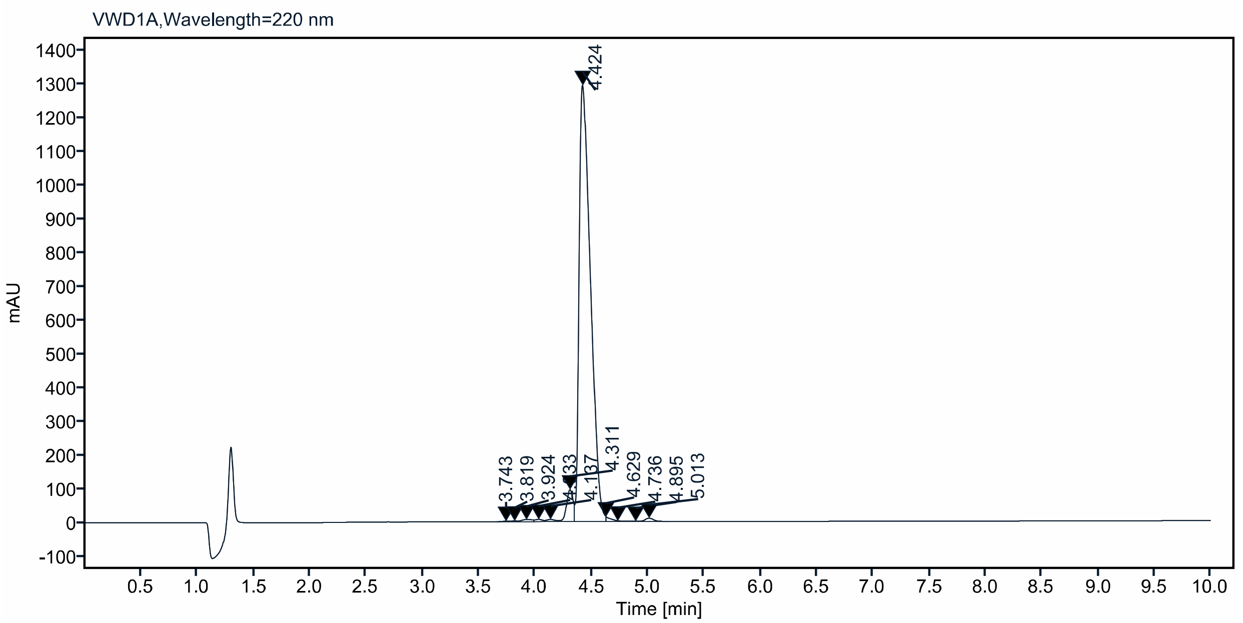

Signal: VWD1A,Wavelength=220 nm

RT [min]	Type	Width [min]	Height	Area	Area%
3.743	BV	0.107	1.342	4.767	0.049
3.819	VV	0.084	2.373	9.590	0.099
3.924	VV	0.142	6.026	41.404	0.427
4.033	VV	0.095	6.879	31.282	0.323
4.137	VV	0.124	6.701	36.353	0.375
4.311	VV	0.140	94.852	374.041	3.862
4.424	VF	0.280	1293.986	9074.607	93.695
4.629	VF	0.107	14.771	45.827	0.473
4.736	VV	0.096	2.288	7.461	0.077
4.895	VV	0.086	1.207	5.634	0.058
5.013	VBA	0.272	10.008	54.273	0.560

Data file: D:\DATE\221111\221111-1 2022-11-11 09-06-28\951207F.D 11/11/2022 2:34:09 PM

Sample name: 951207

Description: Lot#:CV-11-00091
Sample Type:Final

Acq. method: 290- 2000.M

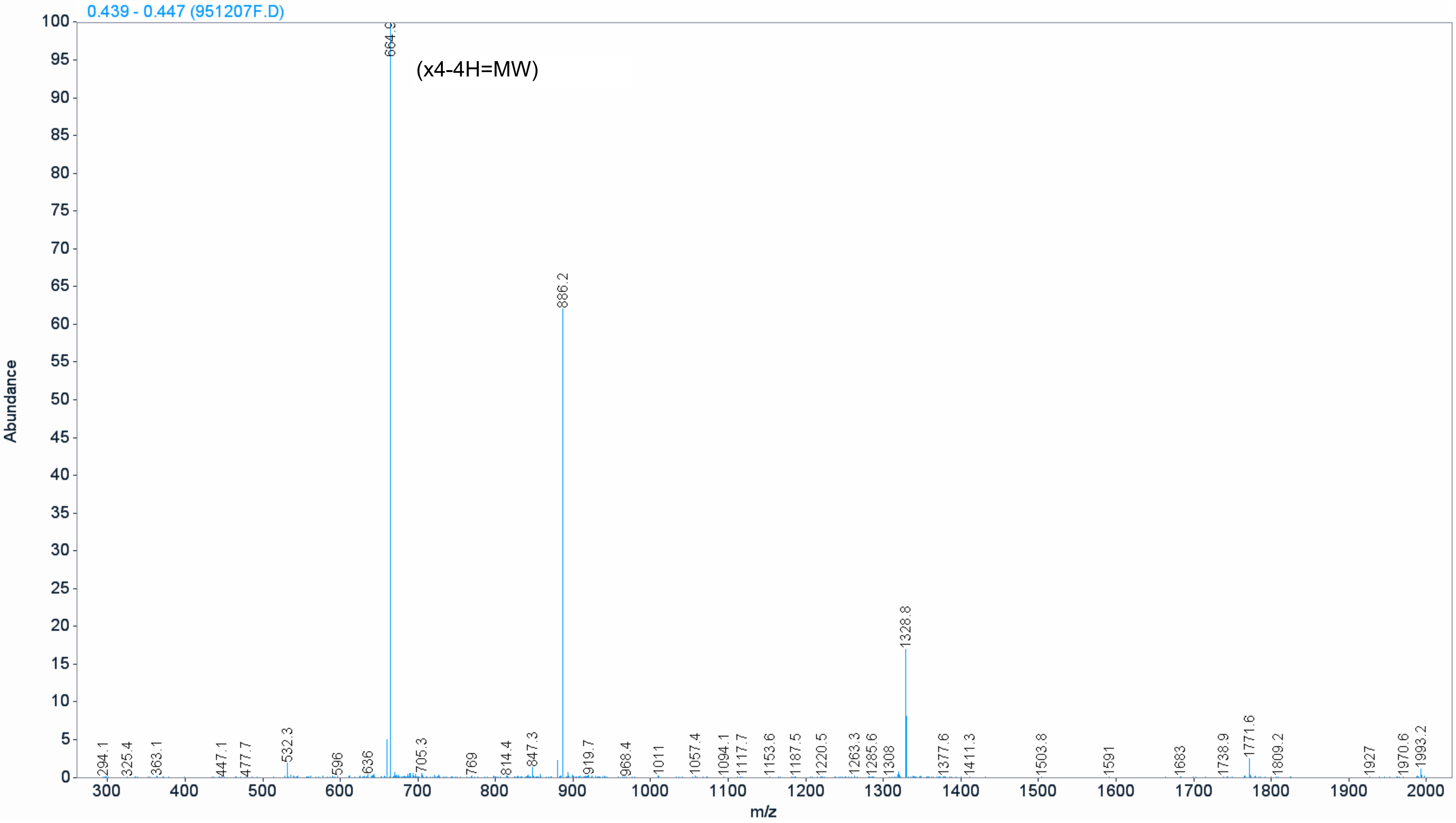

Certificate of Analysis

**Product Name:**	Peptide 6		
**Product #:**	951208	**Lot #:**	CV-10-00448
**Sequence:**	Lys-Ser-Pro-Asp-Asp-Pro-Ser-Cit-Tyr-Ile-Thr-Pro-Asp-Gln-Leu-Ala-Asp-Leu-Tyr-Lys-Ser-Phe-Val		
**Molecular Weight:**	2656.9		
**Mass Spectral Analysis:**	Electrospray "Exhibits correct MW" (see attached MS spectrogram)		
**HPLC Analysis:**	Peptide purity: 95.5% (see attached RP-HPLC chromatogram)		
**Solubility:**	1 mg/ml in water		
**Appearance:**	White lyophilized powder		
**Counter Ion:**	Trifluoroacetate		

Cert. of Analysis Remarks:

Remarks: **Not for human use, research purpose only.**

Quality Assurance By: Ing. Wu **Date: 06/01/23**
Quality Control Department

CPC Scientific Inc.
160 E. Tasman Dr., Suite 200 San Jose, CA 95134 USA
Tel: 1-408-734-3800 or 1-877-272-7241 (Toll-free)
Fax: 1-408-734-3810 or 1-877-272-7244 (Toll-free)
E-mail: sales@cpcscientific.com

Injection Data File Directory	/2022/2021062802/Results/221031/001.rslt
Sample Description	Sample ID:951208 Lot#:CV-10-00448<br>Mobie Phase:A:0.1%TFA in H2O<br>B:0.09%TFA in (80%ACN+20%H20)<br>Flow:1.0ml/min 37.0%-57.0% B buffer in 10min<br>Column:Phenomenex-C18(2) luna 3um 100A 4.6*100mm A2118# 50C

Injection Acquired Date	10/31/2022	Sample Vial Number	P1-A2
Injection Acquired By	sherry song	Injection Volume	12.000
Instrument Name	2021062802	Injection Volume Unit	µL

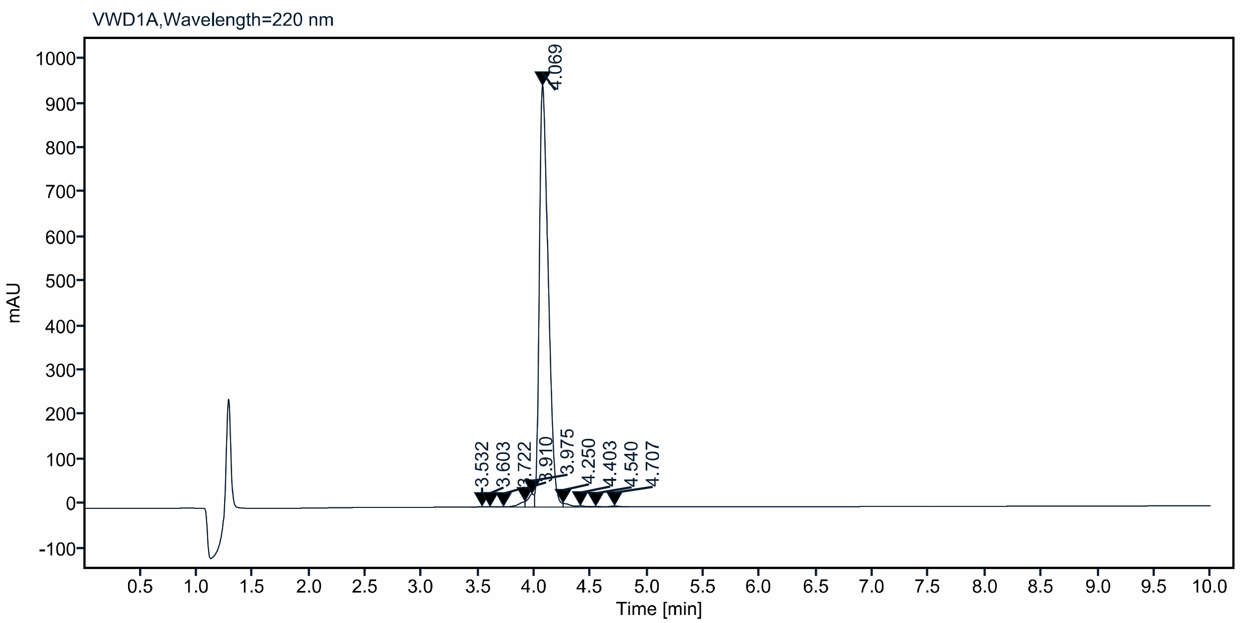

Signal: VWD1A,Wavelength=220 nm

RT [min]	Type	Width [min]	Height	Area	Area%
3.532	BV	0.142	1.198	5.788	0.105
3.603	VV	0.095	1.064	5.099	0.093
3.722	VV	0.077	0.754	3.222	0.059
3.910	VV	0.166	12.159	47.804	0.870
3.975	VV	0.087	29.564	116.328	2.118
4.069	VF	0.253	946.240	5248.816	95.575
4.250	VV	0.112	8.075	32.721	0.596
4.403	VV	0.121	2.475	12.991	0.237
4.540	VV	0.136	1.130	7.112	0.130
4.707	VBA	0.218	2.025	11.969	0.218

Data file: D:\DATE\221031\221031-1 2022-10-31 09-05-28\951208F.D 10/31/2022 10:47:16 AM

Sample name: 951208

Description: Lot#:CV-10-00448
Sample Type:Final

Acq. method: 290- 2000.M

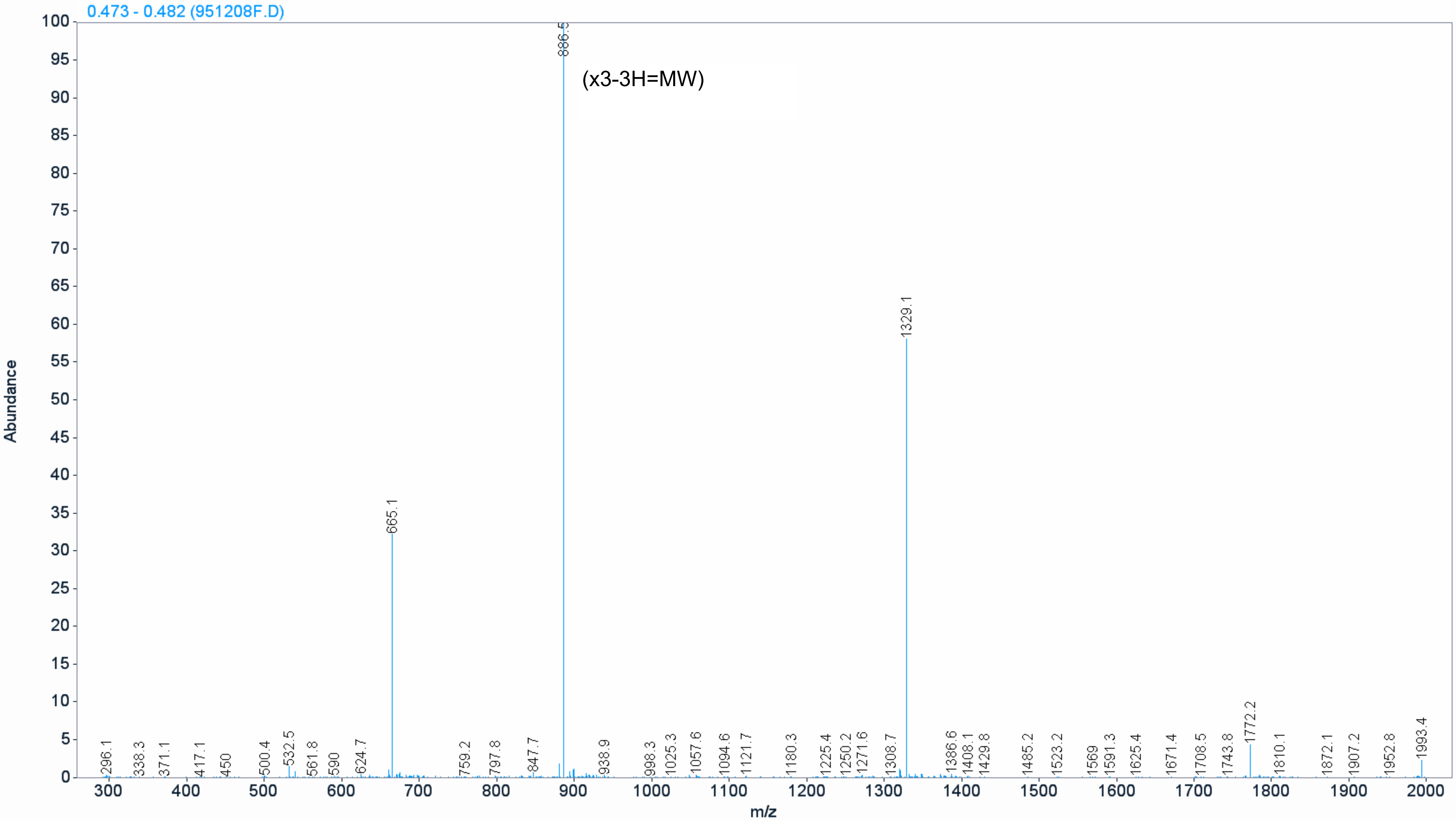

Supplement: Supplementary file 1 [file vaccines-13-00629-s001.zip › Supplementary Figure 1.pdf]
