# Supplementary figures and images for: Citrullinated ENO1 Vaccine Enhances PD-1 Blockade in Mice Implanted with Murine Triple-Negative Breast Cancer Cells
Source: Vaccines (Basel). 2025 Jun 11;13(6):629. doi: 10.3390/vaccines13060629 (PMC12197626; doi:10.3390/vaccines13060629)

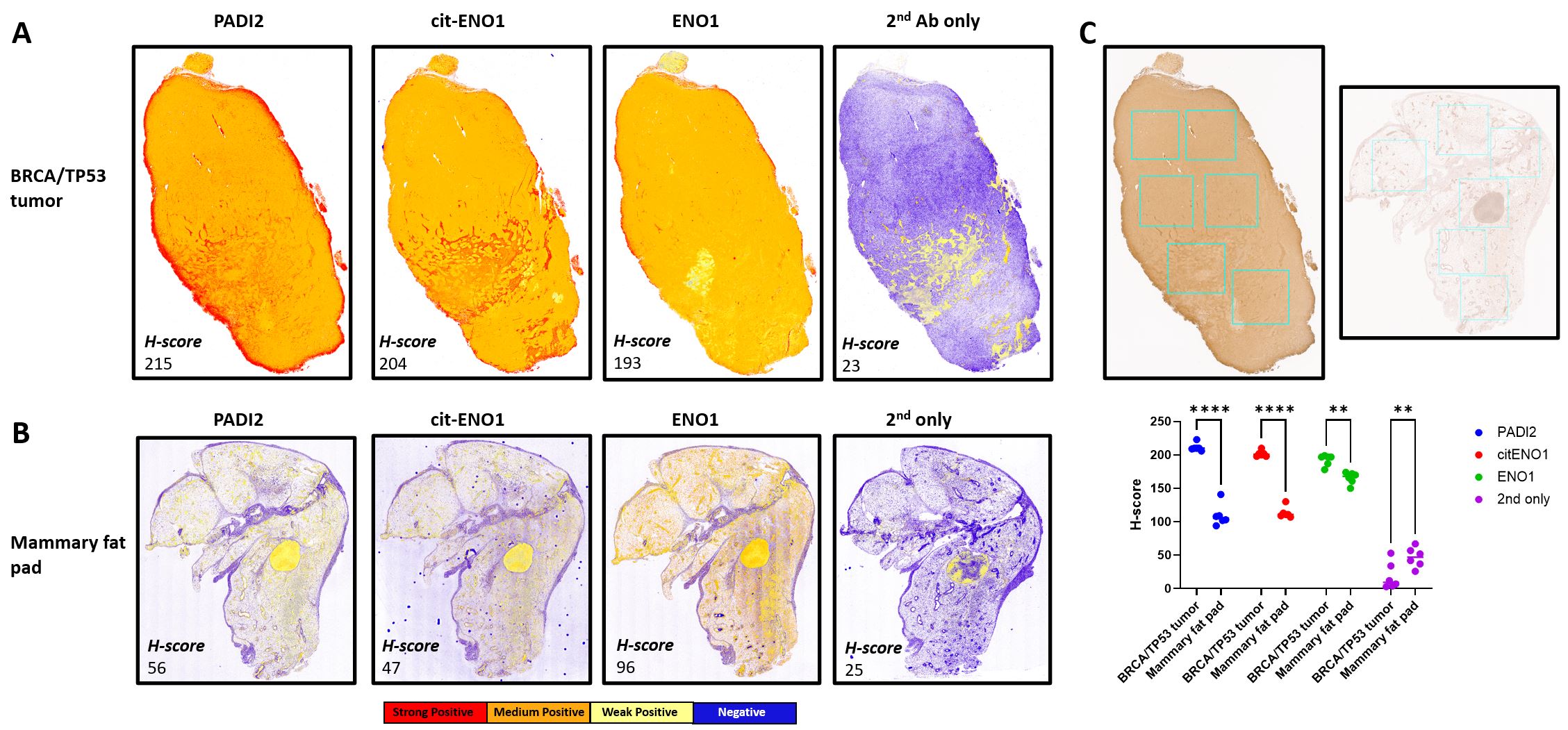

Supplement: Supplementary file 1 [file vaccines-13-00629-s001.zip › Supplementary Figure 2.JPG]

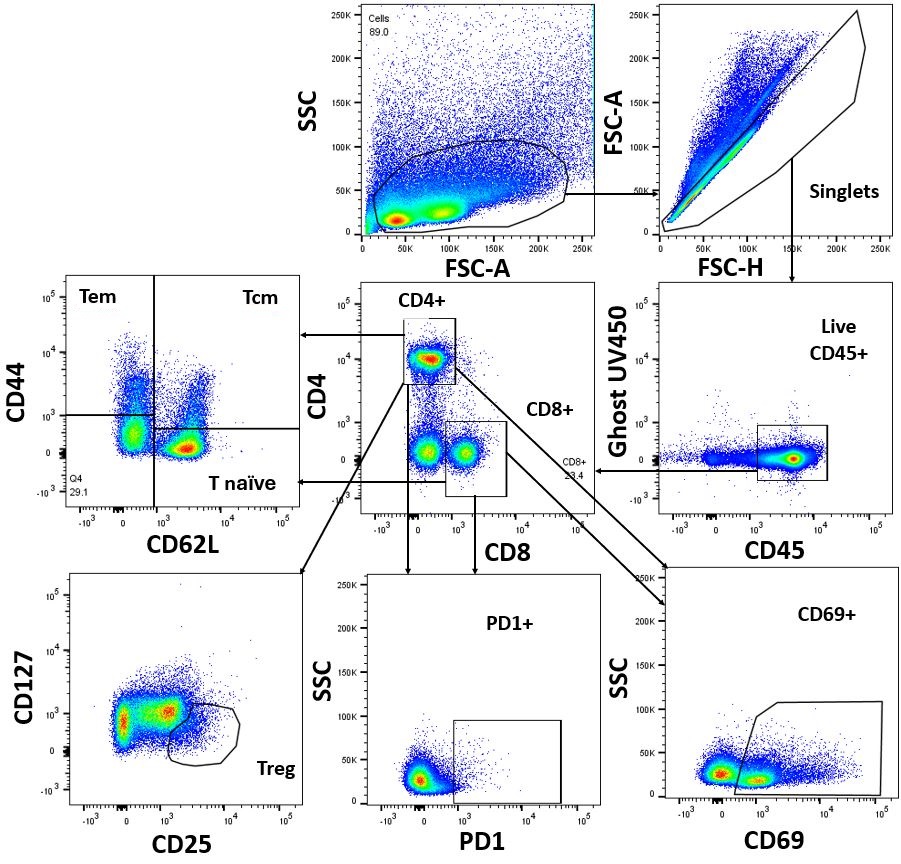

Supplement: Supplementary file 1 [file vaccines-13-00629-s001.zip › Supplementary Figure 3.JPG]

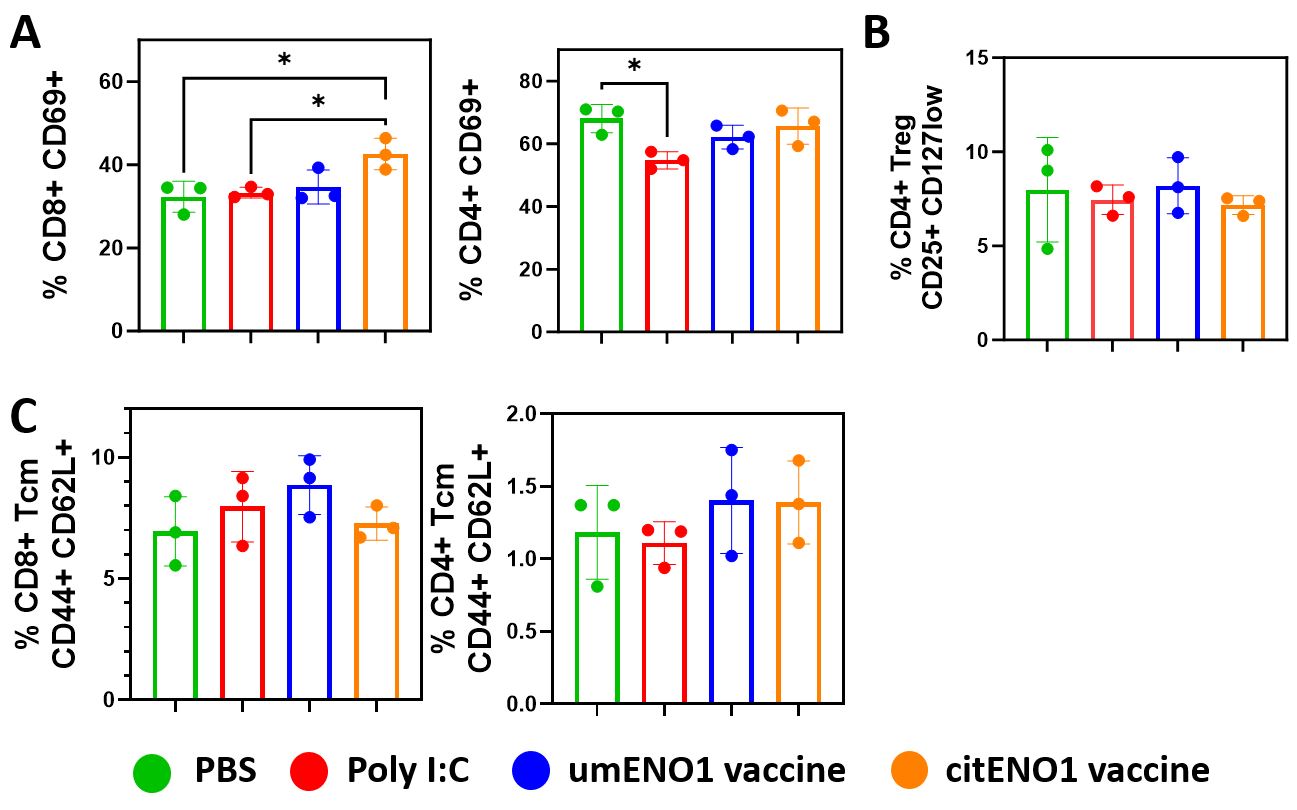

Supplement: Supplementary file 1 [file vaccines-13-00629-s001.zip › Supplementary Figure 4.JPG]

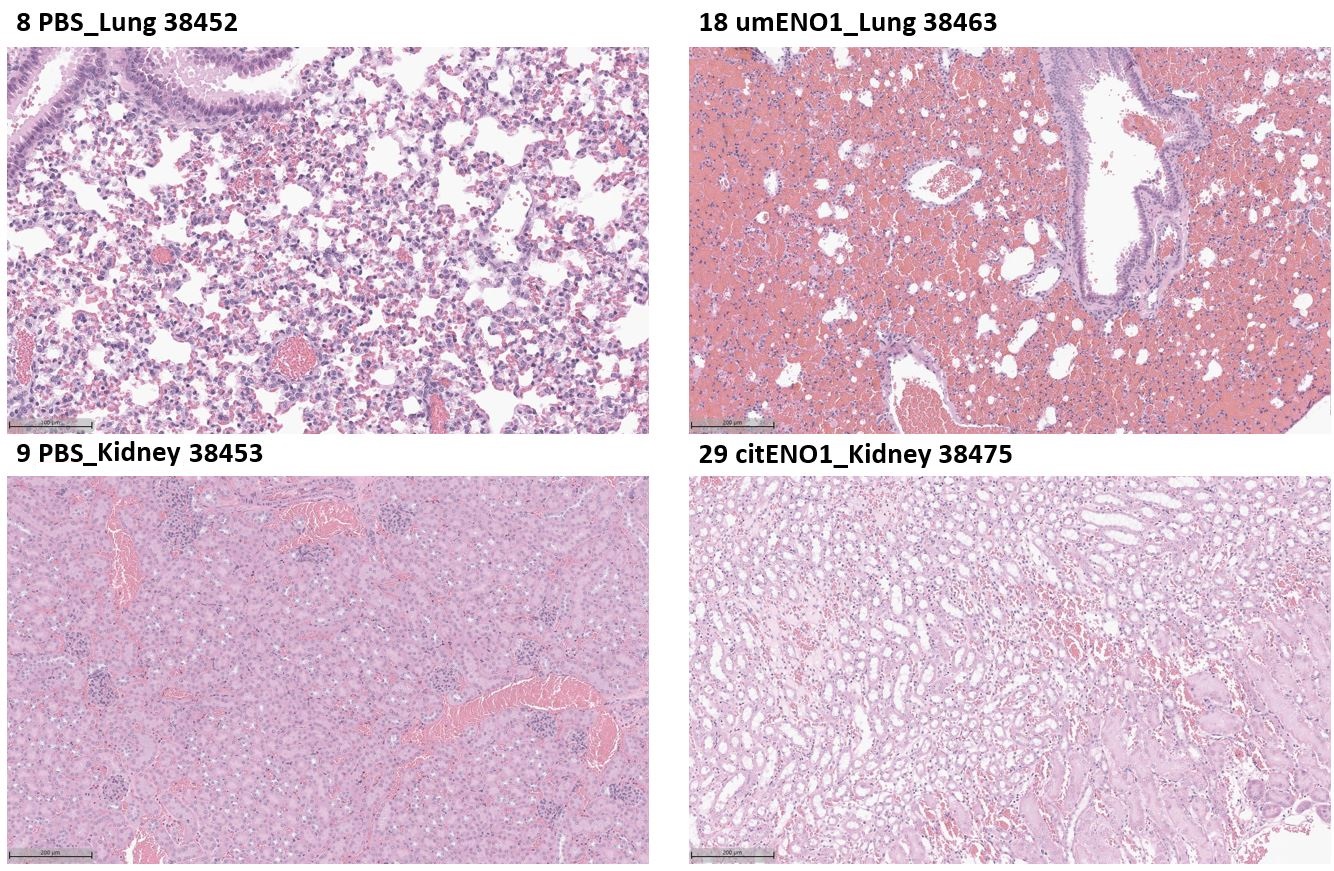

Supplement: Supplementary file 1 [file vaccines-13-00629-s001.zip › Supplementary Figure 5.JPG]

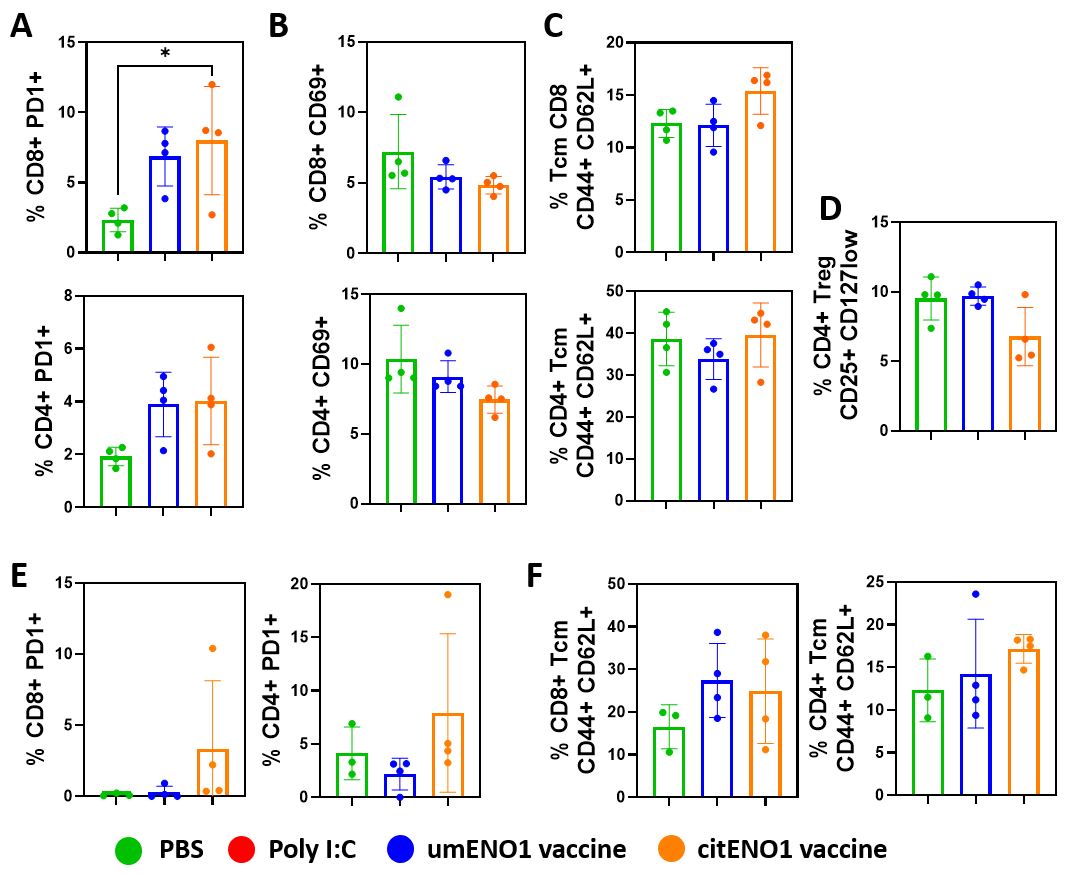

Supplement: Supplementary file 1 [file vaccines-13-00629-s001.zip › Supplementary Figure 6.JPG]

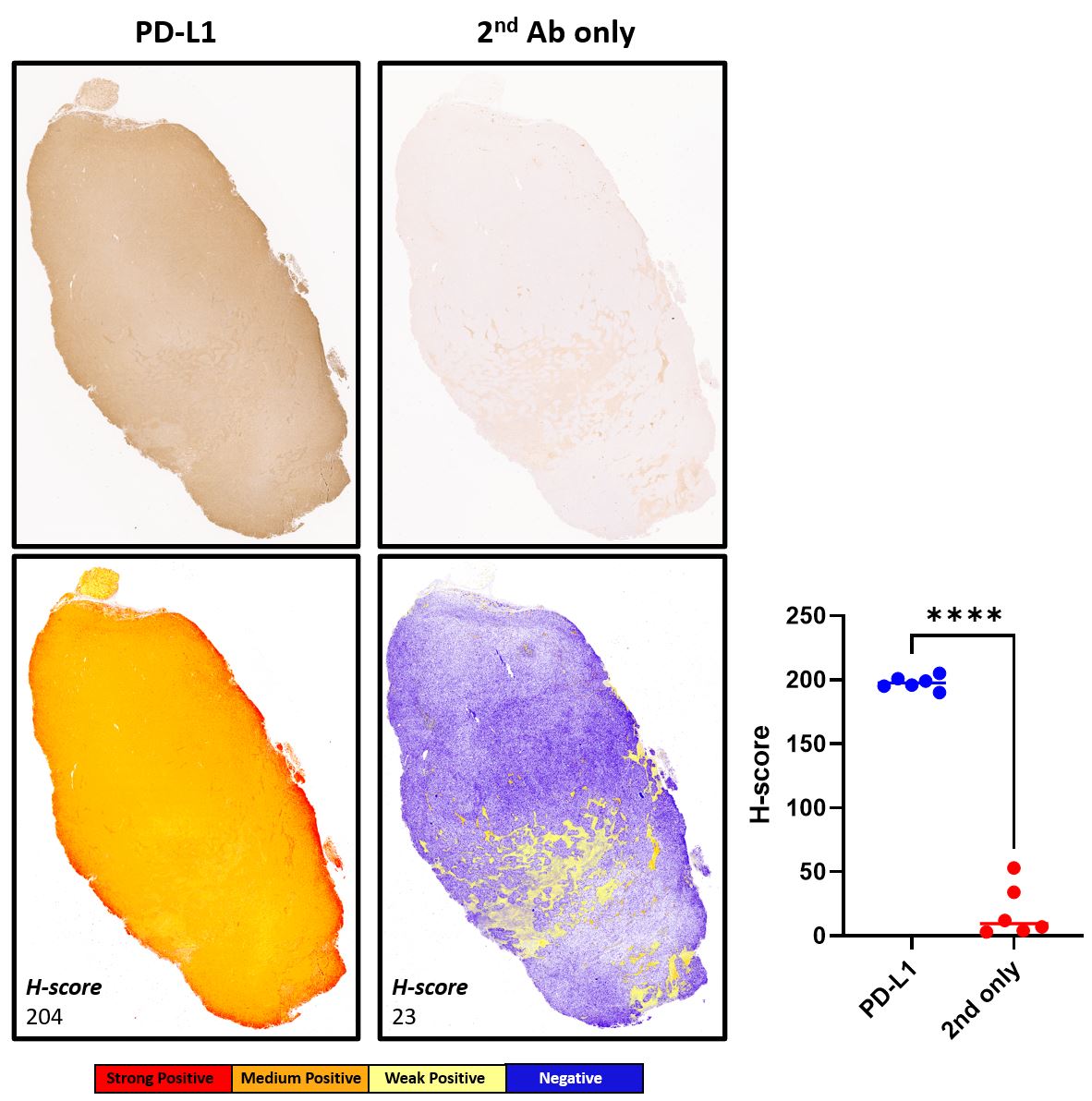

Supplement: Supplementary file 1 [file vaccines-13-00629-s001.zip › Supplementary Figure 7.JPG]

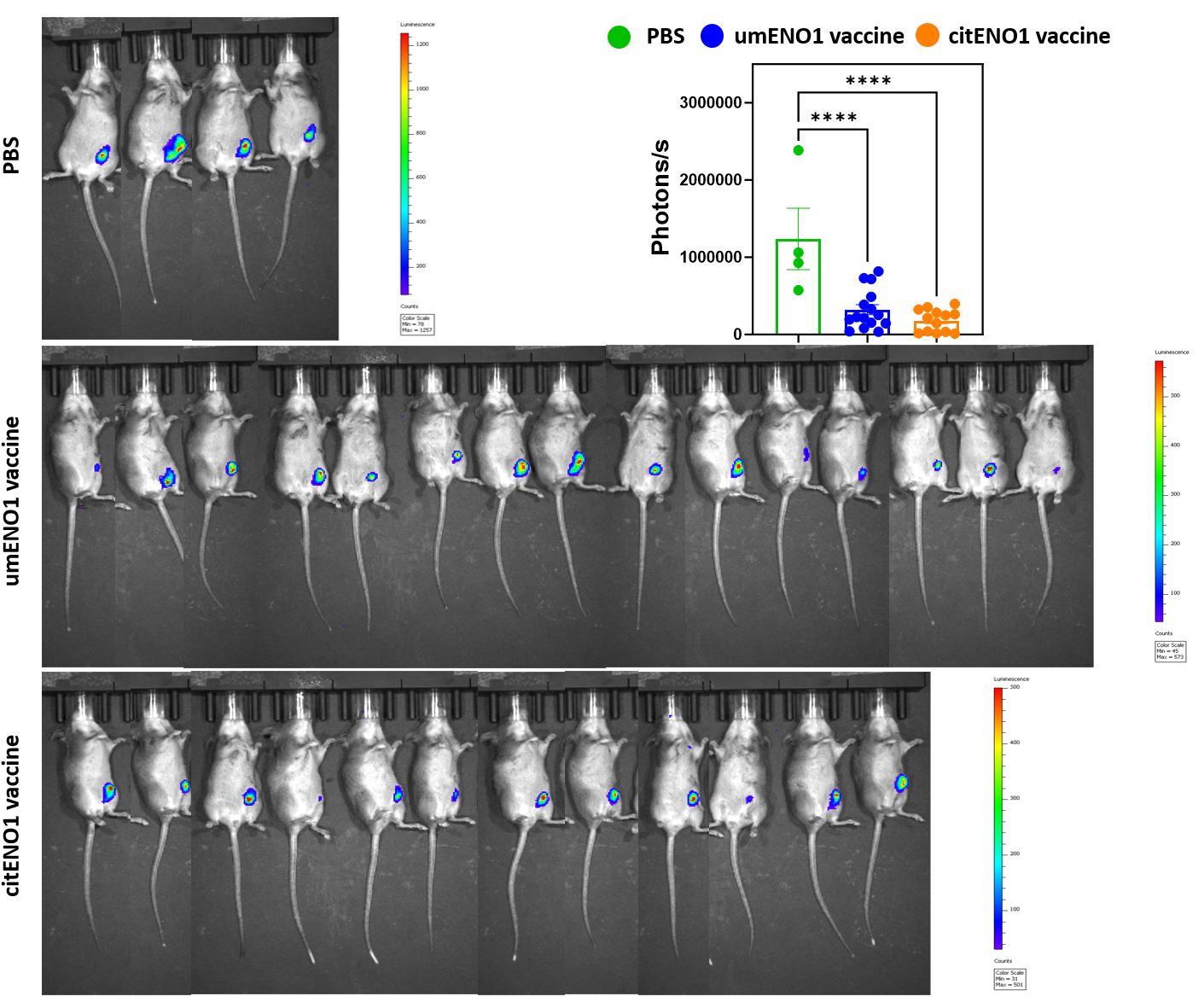

Supplement: Supplementary file 1 [file vaccines-13-00629-s001.zip › Supplementary Figure 8.JPG]
